# The population genetic structure and phylogeographic dispersal of *Nodularia breviconcha* in the Korean Peninsula based on COI and 16S rRNA genes

**DOI:** 10.1371/journal.pone.0288518

**Published:** 2023-07-12

**Authors:** Gyeongmin Kim, Ui Wook Hwang

**Affiliations:** 1 Department of Biology Education, Teachers College & Institute for Phylogenomics and Evolution, Kyungpook National University, Daegu, South Korea; 2 School of Life Sciences, Graduate School, Kyungpook National University, Daegu, South Korea; 3 Department of Biomedical Convergence Science and Technology, School of Industrial Technology Advances, Kyungpook National University, Daegu, South Korea; 4 Institute for Korean Herb-Bio Convergence Promotion, Kyungpook National University, Daegu, South Korea; 5 Phylomics Incorporated, Daegu, South Korea; The Chinese University of Hong Kong, CHINA

## Abstract

A freshwater mussel, *Nodularia breviconcha* (Mollusca: Bivalvia: Unionida) is endemic to Korean Peninsula. It has recently been taxonomically reexamined and elevated from a subspecies of *N*. *douglasiae* to an independent species. But population genetic studies for the species have rarely been conducted. To explore the population genetic structure of *N*. *breviconcha*, the nucleotide sequences of cytochrome oxidase subunit I(COI) and 16S rRNA genes from 135 *N*. *breviconcha* individuals, including 52 from this study and 83 from Choi et al. (2020). We found 23 COI and 11 16S rRNA genes haplotypes. Phylogeny, TCS network, Principal coordinates analysis, and spatial analysis of molecular variance performed with COI gene indicated that there are exist three different genetic lineages in the *N*. *breviconcha* populations: West lineage, Southwest lineage, and Southeast lineage. According to the time calibrated phylogeny, they are likely to be diverged during the late Miocene (8–6 Ma). Geographical distribution patterns of the three genetic lineages may be related to the formation of Taebaek and Sobaek-Noryeong mountain ranges in the Korean Peninsula occurred during the Miocene (30–10 Ma). The present results of this study will be helpful not only for the conservation, but also for the exploration of the population genetic structure of endemic freshwater mussels in the Korean Peninsula.

## Introduction

Unionid freshwater mussels (Mollusca: Bivalvia) are distributed in freshwater systems of all continents except Antarctica [1, 2], and play important roles in the circulation of nutrients, provision of habitat for other living things, and food resources [3, 4]. The order Unionida comprises six families, with approximately 958 species reported to date [5]. Among the six families of the order Unionida, the family Unionidae is the most specious, comprising 753 species and 153 genera [5]. In recent decades, habitats of freshwater mussels have been rapidly destroyed worldwide because of environmental pollution, large-scale river construction and dam construction [6–8], which has led freshwater mussels to become the most endangered taxon [9–12].

The most specious but endangered family Unionidae (Mollusca: Bivalvia: Unionida) is widely distributed in East Asia including the Korean Peninsula [13, 14]. One member of this family, *Nodularia breviconcha* is endemic to the Korean Peninsula and is predominantly observed in the upper and middle streams of rivers [13–15]. In addition to *N*. *breviconcha*, *Nodularia douglasiae* also inhabits the Korean Peninsula. *N*. *breviconcha* has been treated as a subspecies of *N*. *douglasiae* [16]. However, recent phylogenetic and population genetic studies based on Cytochrome c oxidase subunit I gene have continuously demonstrated that *N*. *breviconcha* should be considered an independent species [13, 14, 17, 18]. Lopes-Lima et al. (2014), therefore, provided a new specific name, *N*. *breviconcha* [13]. Choi et al. (2020) conducted a remarkable population genetic study based on inferring the COI and 16S rRNA genes of *N*. *douglasiae* and *N*. *breviconcha* present on the Korean Peninsula, with extensive sampling of *N*. *breviconcha* (biased as this study involved analysis of samples from only two rivers, i.e. Bukhan River and Namhan River). Their results indicate that *N*. *breviconcha* and *N*. *douglasiae* are distantly related genetically and can be established as independent species [14].

*N*. *breviconcha* has morphological characteristics that are similar to those of *N*. *douglasiae*. One notable difference is that shell size of *N*. *breviconcha* is relatively small [13, 15]. Compared to *N*. *douglasiae*, little is known about the biological characteristics of *N*. *breviconcha* such as its detailed geographic distribution and genetic structure. In accordance with Choi et al. (2020), 83 individuals of *N*. *breviconcha* were collected from six rivers, and these contained 16 COI gene haplotypes and five 16S rRNA gene haplotypes. COI and 16S rRNA gene haplotypes in the Bukhan River population exhibited the highest haplotype diversity [14]. The study also briefly discussed the existence of two possible different genetic lineages based on COI gene haplotypes, i.e., the populations of the Bukhan River, Namhan River, and Geum River and the populations of the Seomjin River, Yeongsan River, and Tamjin River. In addition, it was found that the two genetic lineages diverged in the late Miocene (7.35 Ma). However, in Choi et al. (2020), most of the specimens were collected from the Bukhan and Namhan Rivers, and fewer than five samples were collected from the remaining four rivers with no samples collected from the Nakdong River, indicating biased sampling [14]. This resulted in a distinct limitation in accounting for the entire whole population genetic structure of *N*. *breviconcha* in the Korean Peninsula.

Considerable studies have reported the genetic structure of freshwater organisms [14, 19, 20] in the Korean Peninsula. Previous studies on the phylogeographic patterns of the endemic freshwater fish indicated that there were three distinct subdistricts based on this biogeographic discontinuity with spatial separations in the Korean Peninsula, i.e., the west, south, and northeast subdistrict [19, 21]. Population genetics analyses of several freshwater fish [e.g. *Coreoleuciscus splendidus* [22], *Cobitis nalbanti* [23]] indicated that the population genetic structure consistently comprised the three different subdistricts [21, 22]. The main barriers among these subdistricts were found to be the mountain ranges such as the Baekdudaegan and Noryeong/Sobaek mountain ranges [19–21]. In the case of *N*. *douglasiae* [14], there are two genetic lineages in the Korean Peninsula, Clade A (Han River, Nakdong River, and Geum River) and Clade B (Geum River, Yeongsan River, Tamjin River, Seomjin River, and Mangyeong River).

In this study, the population genetic structure and phylogeographic dispersal of *N*. *breviconcha*, were explored by conducting population genetic and phylogeographic analyses based on COI and 16S rRNA gene sequences of 135 *N*. *breviconcha* individuals (newly collected 52 *N*. *breviconcha* from four rivers and 83 *N*. *breviconcha* individuals reported in Choi et al. 2020). The genetic diversity, structure and phylogeographic dispersal of *N*. *breviconcha* populations revealed in the present study may be considered important clues for making successful conservation plans of the species and for helping establish its detailed management units such as defining the evolutionary significant units (ESUs) and management unit (MUs) in the Korean Peninsula [24].

## Results

### Analysis of genetic diversity

**Fig 1:**
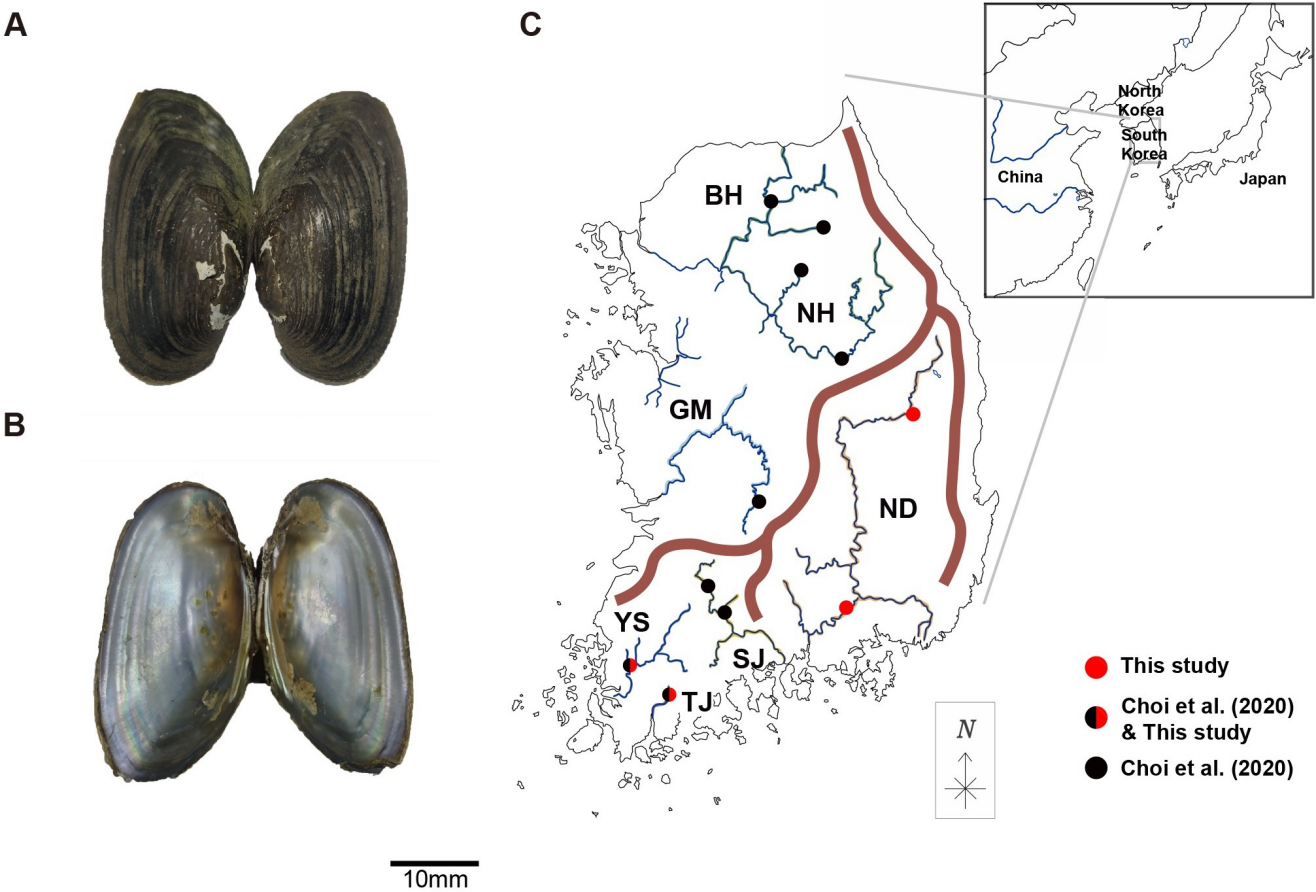
Picture of *Nodularia breviconcha*, and a map showing sample localities for 135 *N*. *breviconcha*. (a-b) The pictures of *N*. *breviconcha* show the (a) external and (b) internal shell morphology. It was photographed by Gyeongmin Kim. These photos were edited using Adobe Illustrator v.22.2 (https://www.adobe.com). (c) The collection sites of 135 *N*. *breviconcha* in the seven river systems of the Korean Peninsula. Each locality is depicted as black, red, or half black and a half red circle, indicating the locality from where the samples were newly collected in this study (red circle), previously collected by Choi et al. (2020) (black circle) and collected from both Choi et al. (2020) and in this study (circle with half black and half red). The full names and abbreviations of the sample localities are as follows: Bukhan River (BH), Namhan River (NH), Geum River (GM), Nakdong River (ND), Seomjin River (SJ), Yeongsan River (YS), and Tamjin River (TJ). Detailed information regarding the locality is presented in S1 Table. The maps are from a free map providing site (https://d-maps.com), which is modified with Adobe Illustrator v.22.2 (https://www.adobe.com).

First, 524 bp fragments of mitochondrial COI gene were amplified and sequenced sufficiently from 52 *N*. *breviconcha* individuals collected from four rivers, i. e., the Namhan River, Nakdong River, Yeongsan River, and Tamjin River on the Korean Peninsula (Fig 1 and S1 Table). In total, 135 COI gene sequences of *N*. *breviconcha* (52 newly collected samples in this study and 83 reported in Choi et al. (2020)) were used for the present analysis. The names of the seven populations examined here were assigned as BH (Bukham River), NH (Namhan River), GM (Geum River), ND (Nakdong River), SJ (Seomjin River), YS (Yeongsan River), and TJ (Tamjin River) based on the rivers from which they were collected. The result of this exercise revealed 23 haplotypes in *N*. *breviconcha* (S2 and S3 Tables). We identified 29 polymorphic sites among observed haplotypes which contained 17 singleton variable sites, and 12 parsimonious informative sites (S4 Table). We calculated genetic diversity indices for each population as presented in Table 1. In the analyses, GM and SJ were excluded because each population contained only one and two sample(s). The number of COI gene haplotypes per population varied from three in TJ to seven in NH. In case of GM and SJ, one and two haplotypes were indicated for each population. The average haplotype diversity (*h*) and nucleotide diversity (*π*) values were 0.865 and 0.008, respectively. The haplotype diversity was the highest in the BH population (*h* = 0.695). The highest nucleotide diversity was observed in the ND population (*π* = 0.004). There were no significant Tajima’s *D*, Fu’s *F*s, and *R*_2_ values based on the COI gene sequences (Table 1).

**Table 1:** Genetic diversity indices of the COI and 16S rRNA gene haplotypes obtained from 135 individuals of *Nodularia breviconcha* in the Korean Peninsula.

River	Pop	COI										16S rRNA									
		*N*	*N*_1_	*N*_H_	*N*_P_	*h*	*S*	*Π*	Tajima’s *D*	Fu’s *F*s	*R*_*2*_	*N*	*N*_1_	*N*_H_	*N*_P_	*h*	*S*	*π*	Tajima’s *D*	Fu’s *F*s	*R*_*2*_
Bukhan	BH	29	29	6	3	0.695	6	0.002	-1.045	-1.784	0.219	29	29	3	1	0.197	2	0.001	-1.249	**-1.628** ^*^	0.160
Namhan	NH	44	44	7	1	0.553	9	0.002	-1.432	-2.029	0.039	44	44	3	2	0.090	3	0.000	**-1.708** ^*^	**-2.121** ^*^	0.139
Nakdong	ND	27	27	5	4	0.496	11	0.004	-0.992	1.155	0.108	27	27	2	1	0.205	3	0.002	-0.508	2.008	0.150
Yeongsan	YS	19	19	4	4	0.509	4	0.002	-0.296	0.001	0.272	19	18	4	3	0.686	3	0.003	0.427	-0.110	0.163
Tamjin	TJ	13	13	3	2	0.564	3	0.001	0.000	0.693	0.574	13	12	1	0	0.000	0	0.000	0.000	-	0.163
**Total**		135	135	23	23	0.865	29	0.008	-0.480	-3.650	0.383	135	131	11	11	0.678	8	0.003	-0.494	-3.758	0.360

This table includes the abbreviation of the population (Pop), the number of samples (*N*), the number of samples from which PCR fragments were obtained (*N*_1_) the number of haplotypes (*N*_H_), the number of private haplotypes (*N*_P_), haplotype diversity (*h*), the number of segregating sites (*S*), and nucleotide diversity (*π*). Significant value: Bold (*: *p*<0.05, **: *p*<0.01, ***: *p*<0.001), not significant value: Italic, na: not available, nd: not determined.

A 357 bp length of mitochondrial 16S rRNA gene was newly amplified and sequenced from 52 *N*. *breviconcha* individuals (Fig 1 and Table 1). The complete dataset included 131 16S rRNA gene sequences of *N*. *breviconcha*, comprising data from 52 newly collected and 79 samples from Choi et al. (2020). Based on this dataset, 11 haplotypes in 16S rRNA gene were found (S5 and S6 Tables). Eight polymorphic sites from *N*. *breviconcha* were identified, including four singleton variable sites and four parsimonious informative sites (S7 Table). The number of 16S rRNA gene haplotypes per population varied from one in TJ to four in YS. The average haplotype and nucleotide diversity of the entire populations were 0.678 and 0.003, respectively. Both haplotype diversity and nucleotide diversity were the highest in the YS (*h* = 0.686, *π* = 0.003). For TJ, only one haplotype was identified in 14 individuals. The neutrality tests revealed that significant negative values appeared only in NH (Tajima’s *D* and Fu’s *F*s) and BH (only Fu’s *F*s), indicating historical expansions of the population (Table 1).

### Phylogenetic and population genetic analyses

**Fig 2:**
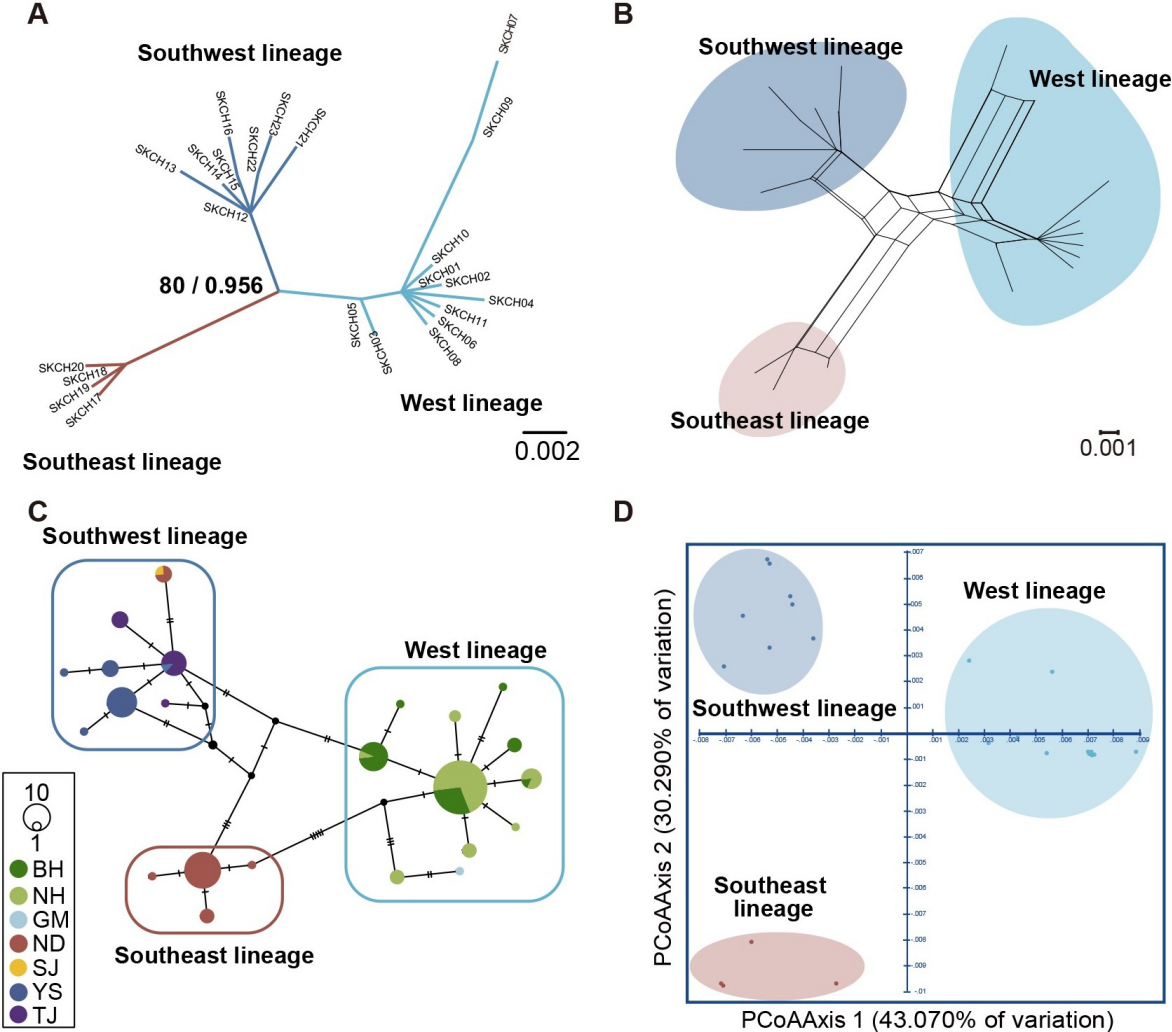
An unrooted maximum likelihood tree, phylogenetic network, TCS network, and PCoA based on 23 COI gene haplotypes from 135 *N*. *breviconcha* individuals inhabiting seven rivers on the Korean Peninsula, indicating the existence of the three different genetic lineages, West, Southeast, and Southwest. (A) An unrooted tree reconstructed maximum likelihood algorithm with 23 COI gene haplotypes. It shows the three different genetic lineages, i. e., West, Southwest, Southeast lineages. The West lineage likely originates from populations inhabiting the Bukhan River, Namhan River, and Geum River. The Southwest lineage is likely from the populations inhabiting a range of Seomjin River, Yeongsan River, and Tamjin River. The Southeast lineages are likely from the populations inhabiting a range of Nakdong River. The numbers of branches indicated node confidence values: BP in ML, and BPP in BI, in order. (B) A phylogenetic network was reconstructed using the Neighbor Net algorithm without an outgroup, showing three different genetic lineages for *N*. *breviconcha* inhabiting the Korean Peninsula, i.e., West, Southeast, and Southwest lineages. (C) A TCS network showing three distinct genetic clusters, corresponding to West, Southeast, and Southwest lineages presented in the (A) unrooted phylogenetic tree, and (B) phylogenetic network. The haplotype frequency is displayed by the circle sizes. (D) A two-dimensional PCoA plot showing the three distinct genetic groups corresponding to the west, southeast, and southwest lineages. The score on the first two axes (Axis 1 = 43.07%, and Axis 2 = 30.29%) from the matrix of genetic distances estimated with the 23 COI gene haplotypes are indicated.

A total of 23 COI gene haplotypes of *N*. *breviconcha* were used for reconstructing the maximum likelihood (ML) phylogenetic tree (Fig 2A), which showed that the COI gene haplotypes were divided into three genetic lineages with high nodal support values (BP 80% and BPP 0.960). The unrooted phylogenetic network, TCS network, and PCoA (Fig 2B–2D) also supported the three different genetic lineages presented in the ML tree (Fig 2A). Based on the TCS network (Fig 2C), the three genetic lineages were separated with four to six mutation steps.

On the other hand, based on the 11 haplotypes of 16S rRNA gene, the ML tree, the unrooted phylogenetic network, TCS network, and PCoA (S1 Fig) did not demonstrate the three genetic lineages shown in the results of COI gene (Fig 2). In addition, we performed the phylogenetic and population genetic analyses based on 26 haplotypes that combine COI and 16S rRNA gene sequences (S2 Fig), which showed the same results based on COI gene (Fig 2).

### Population genetic structure

As presented in Table 2A, the pairwise *F*_*ST*_ values among the five populations based on the COI gene sequences of *N*. *breviconcha* were estimated to range from 0.144 (BH and NH) to 0.838 (NH and YS), and all values were significant (Table 2A). Based on the 16S rRNA gene sequences from 131 *N*. *breviconcha* individuals, the values ranged from 0.017 (BH and NH) to 0.897 (NH and TJ) (S8 Table) with non-significant value in BH and NH, BH and YS, NH and YS, and ND and YS. With these correlations, the SAMOVA test for COI gene revealed progressively increasing *F*_*CT*_ values along with an increasing number of groups (K- values; Table 3). For the three groups, the clearest increase in *F*_*CT*_ values and decrease in *F*_*SC*_ were observed, consistent with the number of genetic lineages identified in the phylogenetic tree, unrooted phylogenetic network, TCS network, and PCoA. By combining these results, the names of three genetic lineages were assigned based on geographic distribution patterns, i.e., West lineage (BH, NH, and GM). Southwest lineage (SJ, YS, and TJ), and Southeast lineage (ND). Based on the SAMOVA AMOVA analyses were conducted, which accounted for 75.590% of the variation among the three groups, 7.130% among populations within groups, and 17.280% within populations (S9 Table). In addition, pairwise *F*_*ST*_ values ranged from 0.736 (Southeast and Southwest) to 0.799 (West and Southeast), which was significant (*p* < 0.001; Table 2B). In summary, the results of the AMOVA and *F*_*ST*_ calculations based on the COI gene suggested distinct genetic differences among the three genetic lineages, as shown in the previous results (Fig 2). No significant differences were detected in any distinct group for 16S rRNA gene (S10 Table).

**Table 2:** Pairwise *F*_*ST*_ values estimated with 135 COI gene sequences from *N*. *breviconcha* (A) among the seven populations and (B) three genetic lineages defined by SAMOVA in the Korean Peninsula.

**A**							
Pop	BH		NH		ND	YS	TJ
BH	0						
NH	**0.144** ^***^		0				
ND	**0.789** ^***^		**0.810** ^***^		0		
YS	**0.832** ^***^		**0.838** ^***^		**0.745** ^***^	0	
TJ	**0.829** ^***^		**0.832** ^***^		**0.743** ^***^	**0.418** ^***^	0
**B**							
Lineage		West (BH-NH-GM)		Southwest (SJ-TJ-YS)		Southeast (ND)	
West (BH-NH-GM)		0					
Southwest (SJ-TJ-YS)		**0.788** ^***^		**0**			
Southeast (ND)		**0.799** ^***^		**0.736** ^***^		**0**	

Statistically significant values are written in bold: *P < 0.05; **P<0.01; ***P<0.001.

**Table 3:** Spatial analysis of molecular variance (SAMOVA) performed based on the COI gene sequences of 135 individuals with the highest *F*_*CT*_ (maximum variation between defined populations).

K value	*F*_*CT*_	*F*_*SC*_	*F*_*ST*_	Grouping
2	**0.503** ^*^	**0.655** ^***^	**0.828** ^***^	(BH-NH-GM)(ND-SJ-TJ-YS)
3	**0.756** ^*^	**0.292** ^***^	**0.827** ^***^	(BH-NH-GM)(ND)(SJ-TJ-YS)
4	**0.785** ^**^	**0.195** ^***^	**0.827** ^***^	(BH-NH)(GM)(ND)(SJ-TJ-YS)
5	**0.797** ^*^	**0.115** ^***^	**0.821** ^***^	(BH-NH)(GM)(ND)(SJ-TJ)(YS)
6	0.798	**0.110** ^***^	**0.820** ^***^	(BH-NH)(GM)(ND)(SJ)(TJ)(YS)

Statistically significant values are written in bold: *P < 0.05; **P<0.01; ***P<0.001.

### Analysis of demographic history and divergence time estimation

**Fig 3:**
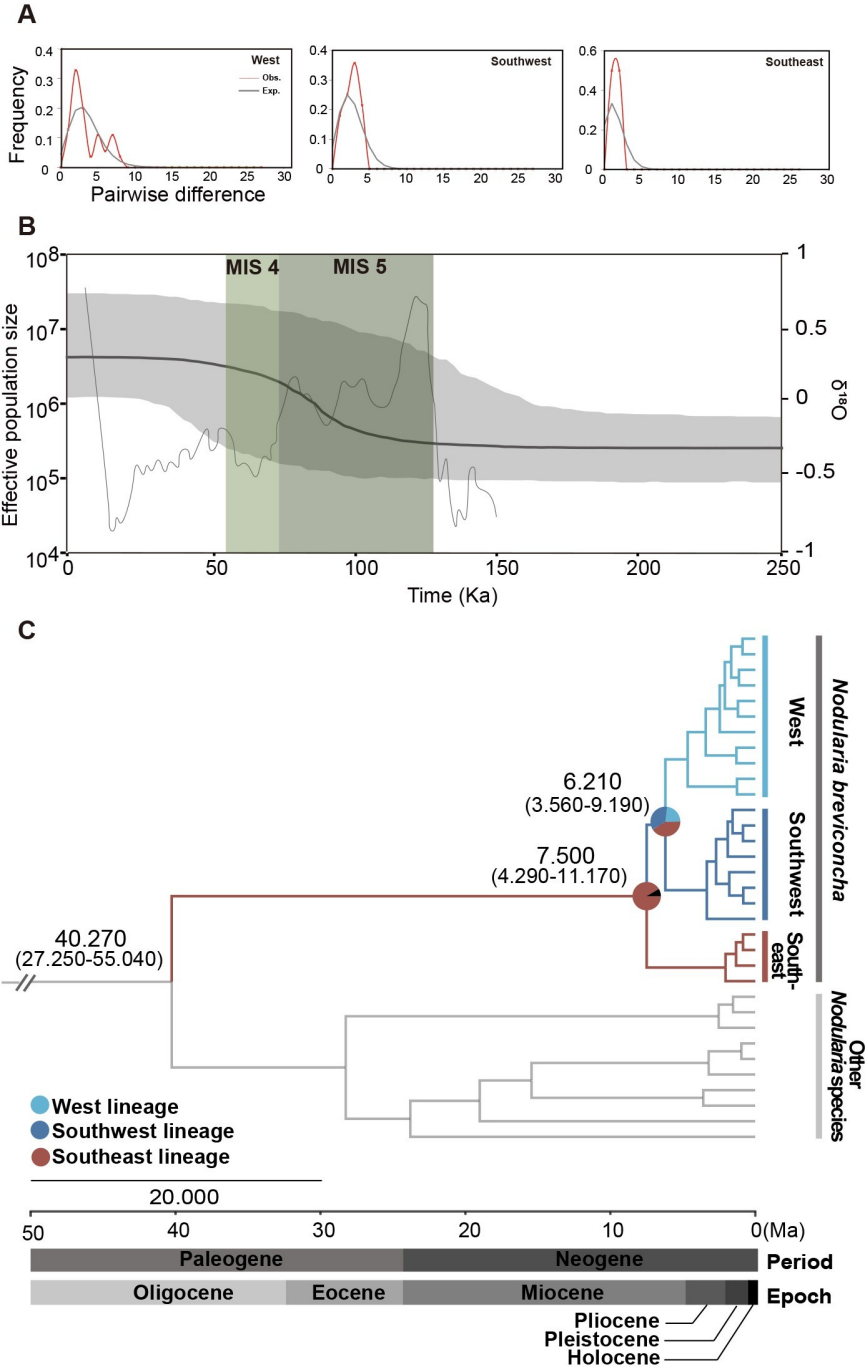
Mismatch distribution analyses (MMD), Bayesian skyline plots (BSPs), and time-calibrated Bayesian trees of COI gene haplotypes of *N*. *breviconcha*. (A) MMD result in a multimodal curve for West lineage, and an unimodal curve for Southwest and Southeast lineages. Gray and red solid lines represent the expected distribution of pairwise differences and the observed distribution under a demographic expansion model, respectively. (B) BSP analyses show the fluctuation of effective population size based on 23 COI gene haplotypes of *N*. *breviconcha*. The graph in gray depicts concentration of oxygen isotope (Waelbroeck et al., 2002) during 150 Ka. (C) Time-calibrated Bayesian tree based on 23 COI gene haplotypes of *N*. *breviconcha* based on the strict clock model using the BEAST 2.6.0. program with the inference of ancestral areas the Bayesian binary Markov chain Monte-Carlo (BBM) model using RASP 3.2 program. Ancestral areas were postulated based on the distribution ranges of three genetic lineage of *N*. *breviconcha* shown in the phylogenetic tree. The pie chart on the nodes indicates the probabilities of the ancestral distribution.

Neutrality tests were performed with 23 haplotypes of COI gene and 11 haplotypes of 16S rRNA gene. Both COI and 16S rRNA gene data demonstrated significant negative values for Fu’s *F*s but not for Tajima’s *D* (Table 4). In case of *R*_2_ test, only 16S rRNA gene data calculated significant negative values. Significant negative values were also observed in the results of Fu’s *F*s and *R*_2_ when the tests were measured on three genetic lineages observed in COI gene (Fig 2). In addition, mismatch distribution analyses (MMD) were conducted based on the haplotypes of COI and 16S rRNA genes (Fig 3A and S3 Fig). The result of the COI gene demonstrated multimodal curves, indicating a constant population size, while 16S rRNA gene demonstrated a unimodal curve, suggesting sudden expansion in population. When the analyses were conducted with each of the three genetic lineages observed in COI gene (Fig 3A), only the West lineage resulted in a multimodal curve, whereas the remaining lineages demonstrated unimodal curves. The results of raggedness index (r) and sum of squared deviations (SSD) values supported the mismatch distribution, but only West lineage among the three lineages was statistically significant (S11 Table).

**Table 4:** The results of the neutrality tests with COI and 16S rRNA genes for *N*. *breviconcha* on the Korean Peninsula.

Gene	Detailed group	Haplotype No.*	Tajima`s *D*	Fu`s *F*s	*R*_2_
COI	West	11	-1.219	**-6.157** ^***^	**0.169** ^***^
	Southeast	4	-1.443	**-8.955** ^***^	**0.330** ^*^
	Southwest	8	-0.754	**-2.367** ^**^	**0.202** ^**^
	Total	23	-0.924	**-21.979** ^***^	0.128
16S rRNA	Total	11	-0.612	**-11.355** ^***^	**0.177** ^*^

*For detailed information on the numbers of haplotypes and employed individuals, refer to S3 and S6 Tables. Statistically significant values are indicated in bold: *P < 0.05, **P<0.01, ***P<0.001.

Bayesian skyline plot (BSP) analyses based on the COI and 16S rRNA gene haplotypes were conducted to examine the pattern of fluctuation in the effective population size of *N*. *breviconcha* (Fig 3B and S4 Fig). The effective population size based on *N*. *breviconcha* in COI gene increased from ca. 100 Ka but stopped the increase at approximately ca. 50 Ka (Fig 3B). With respect to the 16S rRNA gene sequence, the effective population size of *N*. *breviconcha* began to gradually increase from ca. 100 Ka, which stopped the increase at approximately ca. 50 Ka (S4 Fig).

Molecular clock analysis based on by the BEAST program (Fig 3C and S5 Fig) estimated that, the divergence time of *N*. *breviconcha* from the other *Nodularia* species was approximately 40.270 Ma (95% HPD: 55.040–27.250 Ma). Within *N*. *breviconcha*, the Southeast lineage first diverged at approximately 7.500 Ma (95% HPD: 11.170–4.260 Ma), and the Southwest lineage and West lineage diverged from each other at approximately 6.210 Ma (95% HPD: 9.190–3.560 Ma). S-DIVA analysis under a Bayesian binary Markov chain Monte-Carlo (BBM) model (Fig 3C) suggested that a hypothetical common ancestor of *N*. *breviconcha* originated around the Nakdong River. This result may also indicate that the region may be regarded as a plausible origin of all the examined *N*. *breviconcha* in the Korean Peninsula, suggesting possibility that the Southeast lineage is an ancestral haplotype (Fig 3C).

## Discussion

This study was designed to explore the population genetic structure of *N*. *breviconcha* endemic to the Korean Peninsula. Phylogenetic and population genetic analyses (Fig 2) demonstrated that there are three distinct genetic lineages of *N*. *breviconcha*, which may be divided by geographical barriers, i.e., West lineage comprising the Bukhan River, Namhan River, and Geum River; Southwest lineage comprising the Seomjin River, Tamjin River, and Yeongsan River; Southeast lineage comprising the Nakdong River. This study determined the existence of the Southeast lineage inhabiting the Nakdong River, an aspect that overlooked by Choi et al. (2020).

BSP analysis (Fig 3) indicated that the effective population size of the entire population of *N*. *breviconcha* (Fig 3B) may have gradually expanded from 125 to 50 Ka. Interestingly, the possible gradual expansion period (125–50 Ka) of the West lineage overlapped with the interglacial stage (130–71 Ka) known as marine isotope stage 5 (MIS 5) in the Late Pleistocene. During this period, the sea level and temperature were similar to or higher than the present [25, 26]. After MIS 5, one of the Quaternary glacial periods (71–57 Ka) known as MIS 4 followed [25–27]. From MIS 4 to the present, the population size of the West lineage did not significantly increase. This indicates that abiotic factors in the interglacial period of MIS 5 may contribute a gradual increase in the population size by acting as suitable conditions for habitat [28]. Moreover, there is evidence for the gradual population expansion of the West lineage, a star-like topology in the TCS network (Fig 2C) and negative values in Tajima’s *D* and Fu’s *F*s tests (Table 4).

Molecular clock analysis (Fig 3C) demonstrated that *N*. *breviconcha* may have diverged from a common ancestor of the genus *Nodularia* at ca. 40.270 Ma (95% HPD: 55.040–27.250 Ma), i.e., late Eocene (56.000–33.900 Ma). This was far earlier than the time estimated by Choi et al. (2020) (28.000 Ma; 95% HPD: 12.310–50.520 Ma). The divergence time between the Southeast lineage and West/Southwest lineages in *N*. *breviconcha* was estimated to be ca. 7.500 Ma (95% HPD: 11.170–4.260 Ma) [14]. Subsequently, the West and Southwest lineages diverged at approximately 6.210 Ma (95% HPD:9.190–3.560 Ma). The divergence times for the three genetic lineages can be backtracked to the late Miocene (11.630–5.330 Ma). In particular, the divergence time of the Southwest and West lineages of *N*. *breviconcha* is similar to the 7.350 Ma (95% HPD: 13.230–2.600 Ma) expected by Choi et al. (2020), except for the Southeast lineage for which this divergence time did not apply [14].

Plausibly, the diversification of the three genetic lineages of *N*. *breviconcha* may be related to the mountain ranges located at the boundary of their geographic distribution in the Korean Peninsula. It is most likely that Taebaek and Sobaek mountains may serve to create a geographical boundary separating the Southeast lineage from the West/Southwest lineages. The West and Southwest lineages were later diverged by possible geographical boundaries like the Sobaek‒Noryeong mountains. It is known that the mountain ranges in the Korean Peninsula were formed by long-term uplift after the opening of East Sea during the Miocene [19, 23, 29–31], presumably at approximately 30–10 Ma [32]. Considering the estimated genetic lineage divergence times of *N*. *breviconcha* (ca. 7.500 Ma between southeast and West/Southwest and ca. 6.210 Ma between West and Southwest), it is most likely that the diversification of the three genetic lineages of *N*. *breviconcha* may have occurred along with the geographical isolation caused by the formation of mountain ranges (30–10 Ma) in the Korean Peninsula.

**Fig 4:**
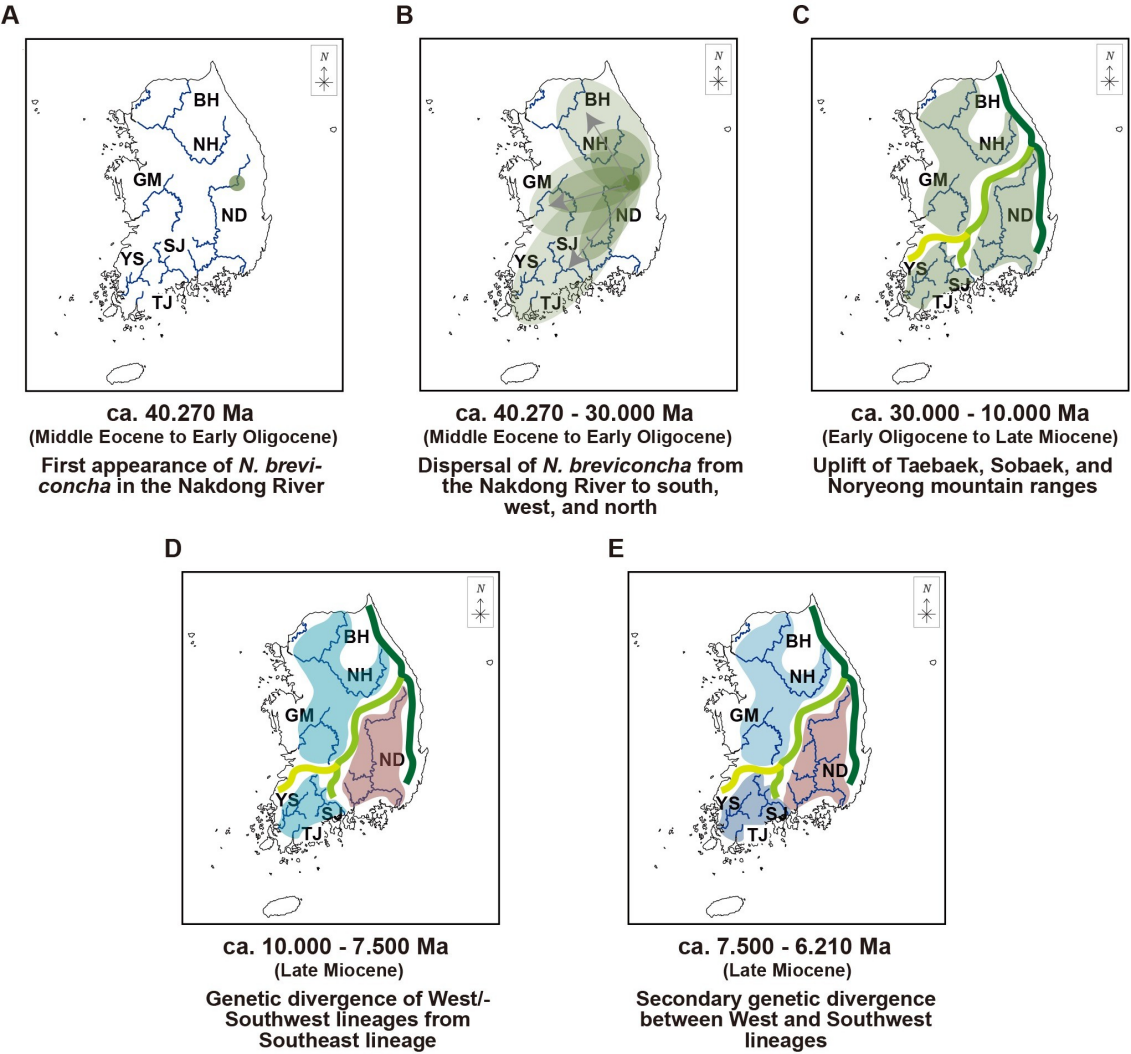
The phylogeographic dispersal scenario of changes of distributions of *N*. *breviconcha*. This scenario of changes of *N*. *breviconcha* is inferred from our genetic results including the time-divergence tree, genetic structure analysis, and previous geological studies. The maps are from a free map providing site (https://d-maps.com), which is modified with Adobe Illustrator v22.2.

Molecular clock, and S-DIVA results of COI gene data from *N*. *breviconcha* endemic in the Korean Peninsula indicate that *N*. *breviconcha* may have first appeared from a certain ancestral *Nodularia* species in middle Eocene (around 40.270 Ma) (Fig 4A). Southeast lineage haplotypes may be regarded as the plausible origins of *N*. *breviconcha*, which is concentrated in the Nakdong River. After the first appearance of *N*. *breviconcha* in the Nakdong River, the population of Southeast lineage may have dispersed to the south, west, and north of the Korean Peninsula (Fig 4B). After uplifts of the Taebaek and Sobaek‒Noryeong mountain ranges between early Oligocene and late Miocene (approximately 30.000–10.000 Ma) (Fig 4C), the genetic divergence of the West (GM, BH, and NH)/Southwest (YS, TJ, and SJ) lineages from Southeast (ND) lineage may have occurred in late Miocene at approximately 7.5000 Ma (Fig 4D) due to geographical isolation. Subsequently, the secondary genetic divergence between the West and Southwest lineages appeared to have occurred at approximately 6.210 Ma (Fig 4E).

This study represents an analysis of population genetic structure based on the COI and 16S rRNA genes of *N*. *breviconcha* endemic to the Korean Peninsula. Previous population genetic studies on freshwater organisms in the Korean Peninsula have predominantly explored freshwater fish. Therefore, the present population genetic and phylogeographic studies on freshwater mussels, along with the recent report on *N*. *douglasiae* and *N*. *breviconcha* published by the same research group [14], are likely to have high value in terms of providing different or complementary perspectives to elucidate the population genetic structure and demographic history of freshwater organisms in the Korean Peninsula. Furthermore, the continuous accumulation of such molecular biogeographical information will help trace historical geology events related to the freshwater system of the Korean Peninsula. Finally, genetic information regarding freshwater molluscan populations such as genetic diversity and structure can provide critical bases for successful conservation plans [24]. The three newly recognized genetic lineages of *N*. *breviconcha* will serve as a representative model for establishing detailed conservation units for Korean freshwater organisms.

## Materials and methods

### Sample collection

All necessary permits for sample collection were obtained from National Institute of Biological Resources, Ministry of Environment, South Korea. A total of 52 *N*. *breviconcha* individuals were collected from four (Namhan, Nakdong, Tamjin, and Yeongsan) rivers in the Korean Peninsula from 2020 to 2021(Fig 1 and S1 Table). After collection, all collected samples were fixed in 95% ethyl alcohol and stored at -20°C in the laboratory until DNA extraction.

### PCR amplification and sequencing

Total DNA was isolated from the muscle tissues (foot) using a DNeasy Blood and Tissue Kit (QIAGEN, Valencia, California, USA). The concentration of extracted DNA was evaluated using NanoDrop 2000 (Thermo Fisher Scientific Co, USA) and electrophoresis on 1% agarose gel.

To amplify partial mitochondrial DNA fragments corresponding to COI and 16S rRNA genes, PCR was conducted using universal primer (LCO22me2/HCO700dy2 [33] for COI gene and 16Sar-L-myt/16Sbr-H-myt [34] for 16S rRNA gene) (S12 Table). The reaction mix for the PCR comprised 20–100ng genomic DNA, 10 mM dNTP, 10 pM of each primer, and 0.25 units of Taq DNA polymerase (Solgent Inc, Daejeon, South Korea) in a total volume of 50 μL. The following thermal cycling conditions were used: denaturation at 95°C for 2 min; 35 cycles of 95°C for 20s, 48–50°C for 40s, and 72°C for 1 min, and a final extension at 72°C for 5 min. Subsequently, 1μL of each PCR product was electrophoresed on a 1% agarose gel containing the eco-dye and observed under UV light. The confirmed PCR bands went through a purification process using a QIAquick PCR Purification Kit (QIAGEN Co., USA) and directly sequenced with an ABI Prism 3730 DNA sequencer (PerkinElmer Inc., USA) using a Big Dye Termination Sequencing Kit (PerkinElmer Inc., USA). All novel sequences of COI gene and 16S rRNA gene discovered in this study were deposited under GenBank accession numbers OM283257-OM283263 for COI gene and OM283265-OM283270 for 16S rRNA gene (S3 and S6 Tables).

### Analyses of population genetic diversity and structure

In addition to the sequences of 52 individuals, this study included 83 COI gene and 79 16S rRNA gene sequences that were previously analyzed by Choi et al. (2020). A total of 135 COI gene and 131 16S rRNA gene sequences were included in each dataset. The nucleotide sequences of mitochondrial COI and 16S rRNA genes obtained from *N*. *breviconcha* were aligned using Clustal X2 [35] and BioEdit 7.2.5 [36]. The identification of variable and parsimonious informative sites and the number of haplotypes (*h*) were estimated using DnaSP 6.11 [37]. The number of private haplotypes unique to each population was determined based on the haplotype list generated from DnaSP 6.11.

To detect the population genetic structure, three different approaches were used. First, pairwise *F*_*ST*_ values were calculated to confirm the pattern of population differentiation using the Arlequin 3.5 program [38]. Second, spatial analysis of molecular variance (SAMOVA) was conducted using SAMOVA 2.0 [39] to determine the best number of groups by defining geographically homogeneous populations that maximize *F*_*CT*_ value between K groups. The number of geographic groups, K, was set from two to six and estimations were performed using the Kimura 2-parameters model. Based on defined groups of populations in SAMOVA, we conducted analysis of molecular variance (AMOVA) to investigate the degree of genetic variance among the groups and populations based on kimura-2-parameter distance using the Arlequin 3.5 program.

### Phylogenetic analyses

Phylogenetic analyses were based on sequence alignment sets of 23 COI gene (S1 Data) and 11 16S rRNA gene haplotypes (S2 Data) obtained in this study and Choi et al. (2020). The analyses were performed using ML and Bayesian inference (BI) algorithms. In particular, unrooted tree topology was used to decipher the phylogenetic relationships among the haplotypes based on COI and 16S rRNA genes. Before the phylogenetic analyses, model selection for ML and BI tree was conducted using the IQ-TREE software package (http://www.iqtree.org). As a result, the substitution models HKY+F+I and TPM2+F+I, based on COI gene haplotypes and 16S rRNA gene, respectively, were selected as the best-fit models under the Bayesian information criterion. The ML tree was reconstructed on the IQ-TREE webserver (https://iqtree.cibiv.univie.ac.at), and each sequence dataset was set to 1000 maximum iterations with 1000 replicates. The BI tree was reconstructed using MrBayes 3.22 [40] under two parallel runs for 10 million iterations with a sampling frequency of 1,000 iterations. After determining that the Markov chain Monte Carlo (MCMC) generations reached a stationary level, the initial 20% of the generations were removed as burn-in. For the present phylogenetic analyses, we employed the COI gene sequences from 135 individuals and 16S rRNA gene sequences from 131 individuals inhabiting the Korean Peninsula, which were from the data obtained in this study as well as previously published in Choi et al. (2020). In addition, previously reported data were retrieved from the NCBI GenBank and added to our final nucleotide sequences for COI and 16S rRNA genes, as listed in S3 and S6 Tables.

Additionally, a phylogenetic network and TCS network constructed using SplitsTree 4.1 [41, 42] based on the neighbor-net algorithm, and PopART [43] based on statistical parsimony approach, respectively. PCoA was conducted using the DARwin 6.0.9 program to confirm and visualize the genetic distance among the populations [44].

### Demographic history

Three different approaches were used to estimate demographic changes. First, three neutrality tests were conducted: Tajima’s *D* [45] and Fu’s *F*s [46] via Arlequin 3.5, and Ramos-Onsin’ *R*_2_ [47] tests using DnaSP 6.11 to examine demographic history of the population and evolutionary neutrality based on COI and 16S rRNA unique gene haplotypes of *N*. *breviconcha*. Second, MMD [48] was performed to check the frequency of pairwise differences using DnaSP 6.11. The values of raggedness index (r) and sum of square deviations (SSD) were calculated to investigate if the data is suitable for the population growth-decline model using Arlequin 3.5 program. Third, a BSP was computed using the BEAST 2.6.0 program [49, 50] to examine the historical demographic fluctuation since the time of the most recent common ancestor. The mutation rates of 2.0 x 10^−8^ estimated by Liu *et al*. (2017) [16] was used with HKY substitution model [16]. Total 5 million steps was used, containing sampling process every 1,000 generations in MCMC method, and the calculation of ESS value and construction of the BSP [51] was conducted by TRACER 1.6 program [52].

### Divergence time estimation

The hypothetical divergence time of the nodes in the *N*. *breviconcha* phylogeny was conducted using BEAST 2.6.0. program [49, 50] based on COI gene sequences. As obvious fossil records of the genus *Nodularia* are not available, the fossil calibrations adapted to the analysis were estimated based on the outgroup taxa, Parreysiinae by designating priors for the outgroup taxa using a “Monophyly” option of the BEAUti 2 program as (Parreysiinae, (*Nodularia*)). The calibration age of the node where the subfamily *Parreysiinae* and genus *Nodularia* diverge was referred to Bolotov *et al*. (2017) (158.4 Ma; normal distribution) [3, 53]. Based on this calibration point, divergence estimation was conducted using a calibrated Yule speciation model with a strict clock algorithm [54]. To reconstruct the time-divergence tree, HKY model was used with correlations for the gamma distribution. Posterior distributions of parameters were estimated using 5,000,000 MCMC generations (sampled every 1,000 generations). The first 20–25% of the trees were removed as an appropriate burn-in, and the resultant 3,001 trees were combined into a maximum clade credibility tree using the TreeAnnotator 2.6.0 program [55]. The consensus tree was visualized in the FigTree 1.4.2 program [56]. COI gene sequence information for the outgroups is listed in S13 Table. To estimate the distribution of a hypothetical common ancestor, a BBM [57] was adapted to the BEAST tree that was implemented in the RASP 3.2 program [58]. In addition, for calculating the program, we postulate three distributed areas and coded for each taxa, A) the region of the West lineage including the Bukhan River, Namhan River, and Geum River; B) Southeast lineage including the Nakdong River; C) Southwest lineage including the Seomjin River, Yeongsan River, and Tamjin River. The COI gene sequences of outgroups are listed in S13 Table.

## Supporting information

S1 Fig (A) An unrooted maximum likelihood tree, (B) Phylogenetic network, (C) TCS network, and (D) PCoA based on 11 16S rRNA gene haplotypes from 131 *Nodularia breviconcha* individuals inhabiting the river systems of the Korean Peninsula.
(TIF)

S2 Fig (A) An unrooted maximum likelihood tree, (B) phylogenetic network, (C) TCS network, and (D) PCoA based on 26 haplotypes that combine COI gene and 16S rRNA gene sequences from 131 *N*. *breviconcha* individuals inhabiting seven rivers on the Korean Peninsula, indicating the existence of the three different genetic lineages, West, Southeast, and Southwest.
(TIF)

S3 Fig The mismatch distribution analysis (MMD) estimated based on the (A) 23 COI gene and (B) 11 16S rRNA gene haplotypes of *N*. *breviconcha*. (TIF)

S4 Fig The Bayesian skyline plot (BSP) analysis estimated based on 11 16S rRNA gene haplotypes of *N*. *breviconcha*. (TIF)

S5 Fig Time-calibrated tree reconstructed with 23 haplotypes based on COI gene haplotypes of *N*. *breviconcha* using the BEAST 2.6.0. program. (TIF)

S1 Table List of collection sites and the number of *Nodularia breviconcha* individuals in each of the seven rivers. (DOCX)

S2 Table Summary of sequence information of respective COI gene haplotypes from the *N*. *breviconcha* species in the present analyses. (DOCX)

S3 Table Distribution of 23 COI gene haplotypes found in 135 individuals of *N*. *breviconcha* collected from the seven freshwater systems in the Korean Peninsula. (DOCX)

S4 Table Polymorphic sites in sequences of the 23 COI gene haplotypes of *N*. *breviconcha*. (DOCX)

S5 Table Summary of sequence information of respective 16S rRNA gene haplotypes from the *N*. *breviconcha* species used in the present analyses. (DOCX)

S6 Table Distribution of 11 16S rRNA gene haplotypes found in 131 individuals of *N*. *breviconcha* collected from the six freshwater systems in the Korean Peninsula. (DOCX)

S7 Table Polymorphic sites in sequences of the 11 16S rRNA gene haplotypes of *N*. *breviconcha*. (DOCX)

S8 Table Pairwise *F*_*ST*_ values estimated with the five populations of *N*. *breviconcha* based on 16S rRNA gene sequences in the Korean Peninsula. (DOCX)

S9 Table Analysis of molecular variance (AMOVA) performed based on the COI gene sequences of 135 *N*. *breviconcha* individuals. (DOCX)

S10 Table Spatial analysis of molecular variance (SAMOVA) performed based on the 16S rRNA gene sequences of 131 individuals with the highest *F*_*CT*_ (maximum variation between defined populations). (DOCX)

S11 Table Raggedness index (r) and sum of squares deviation (SSD) estimated from the mismatch distribution analysis based on COI and 16S rRNA genes for *N*. *breviconcha* on the Korean Peninsula. (DOCX)

S12 Table The information of primers used for PCR amplification of COI and 16S rRNA genes. (DOCX)

S13 Table Summary of sequence information of outgroups used in the time-divergence tree using the BEAST 2.6.0. (DOCX)

S1 Data Nucleotide sequence alignment of 23 COI gene haplotypes of *N*. *breviconcha*. (TXT)

S2 Data Nucleotide sequence alignment of 11 16S rRNA gene haplotypes of *N*. *breviconcha*. (TXT)
