# Supplementary figures and images for: The population genetic structure and phylogeographic dispersal of Nodularia breviconcha in the Korean Peninsula based on COI and 16S rRNA genes
Source: PLoS One. 2023 Jul 12;18(7):e0288518. doi: 10.1371/journal.pone.0288518 (PMC10337957; doi:10.1371/journal.pone.0288518)

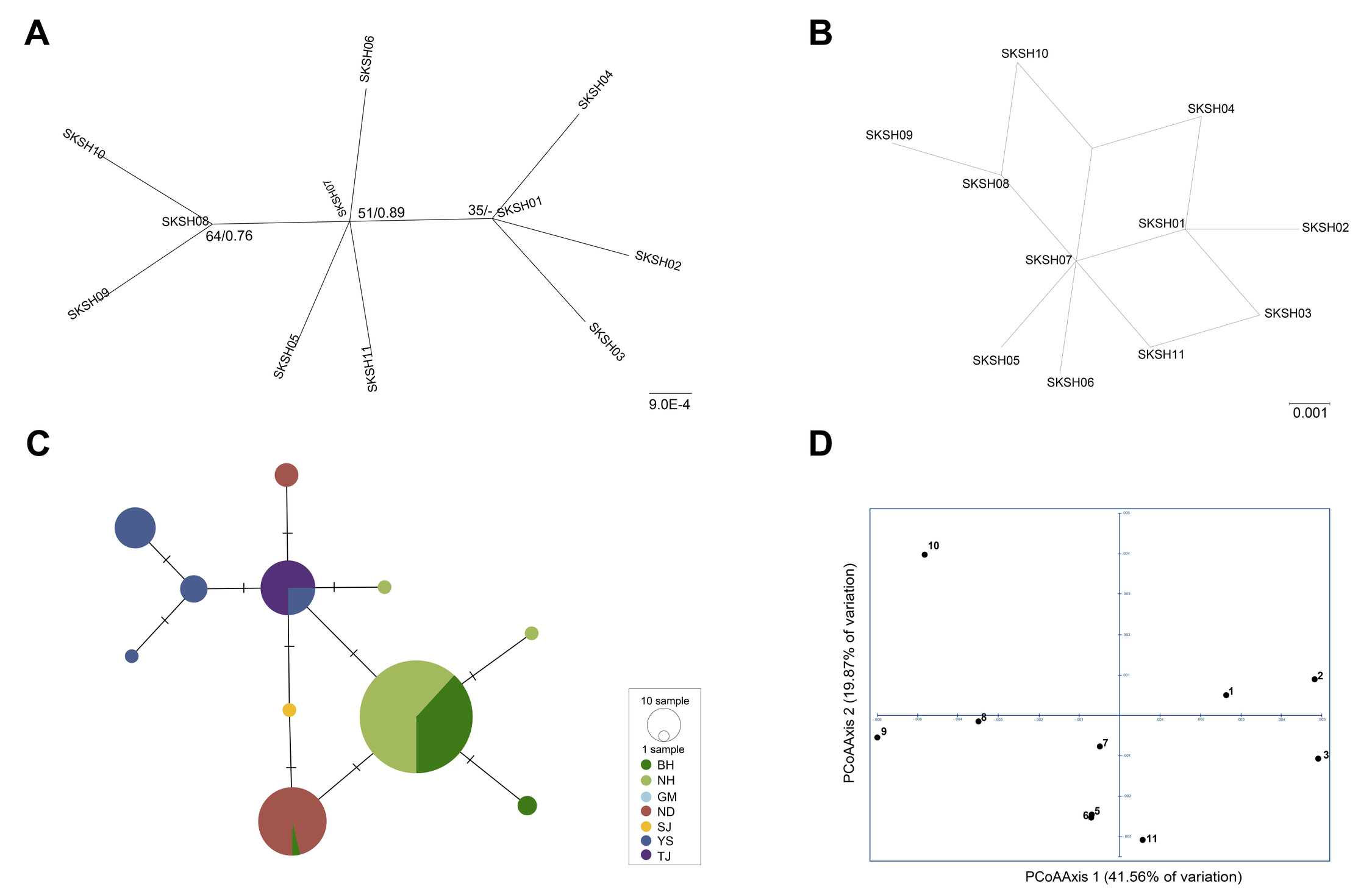

Supplement: S1 Fig — (A) An unrooted maximum likelihood tree, (B) Phylogenetic network, (C) TCS network, and (D) PCoA based on 11 16S rRNA gene haplotypes from 131 Nodularia breviconcha individuals inhabiting the river systems of the Korean Peninsula. (TIF) [file pone.0288518.s001.tif]

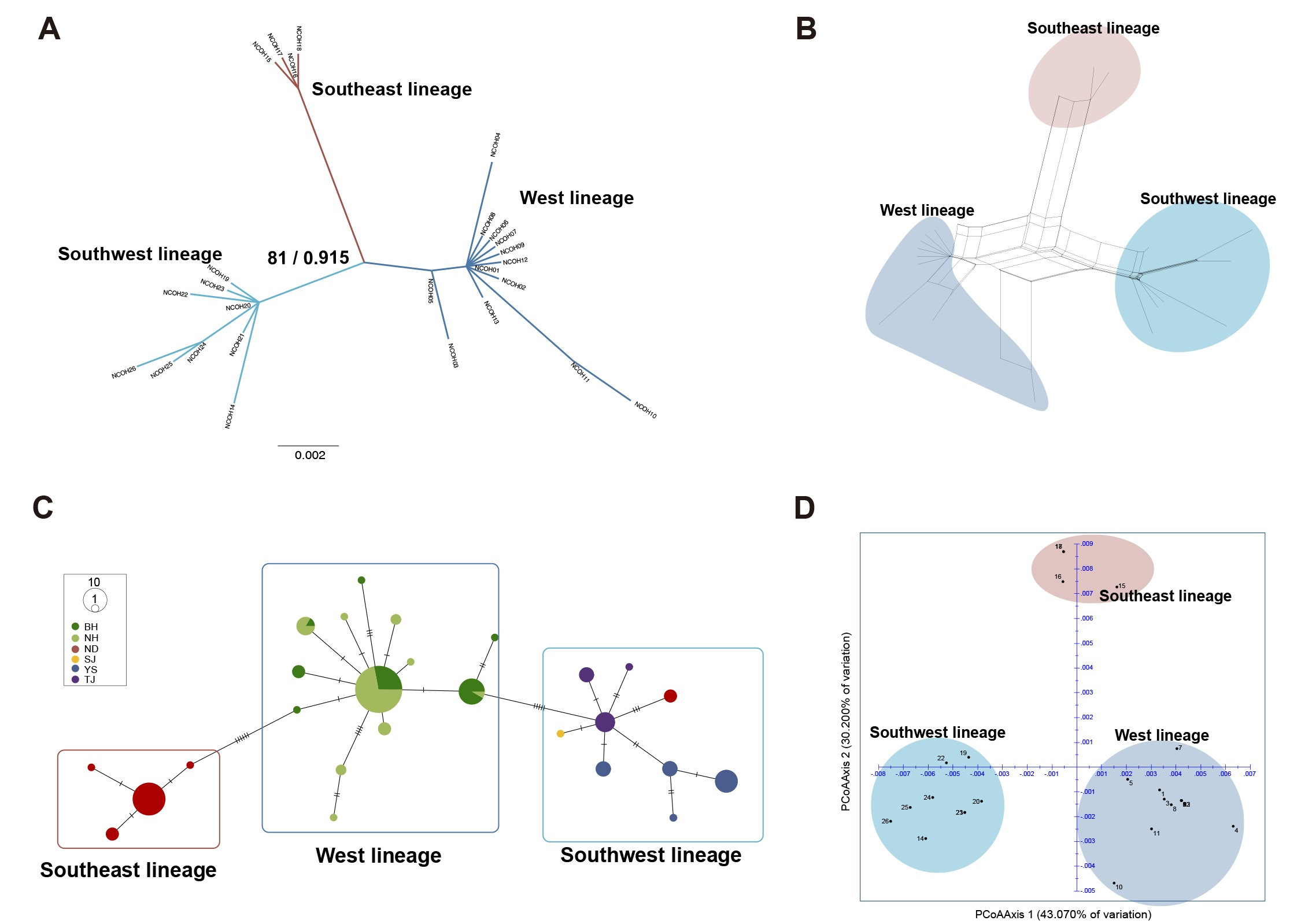

Supplement: S2 Fig — (A) An unrooted maximum likelihood tree, (B) phylogenetic network, (C) TCS network, and (D) PCoA based on 26 haplotypes that combine COI gene and 16S rRNA gene sequences from 131 N. breviconcha individuals inhabiting seven rivers on the Korean Peninsula, indicating the existence of the three different genetic lineages, West, Southeast, and Southwest. (TIF) [file pone.0288518.s002.tif]

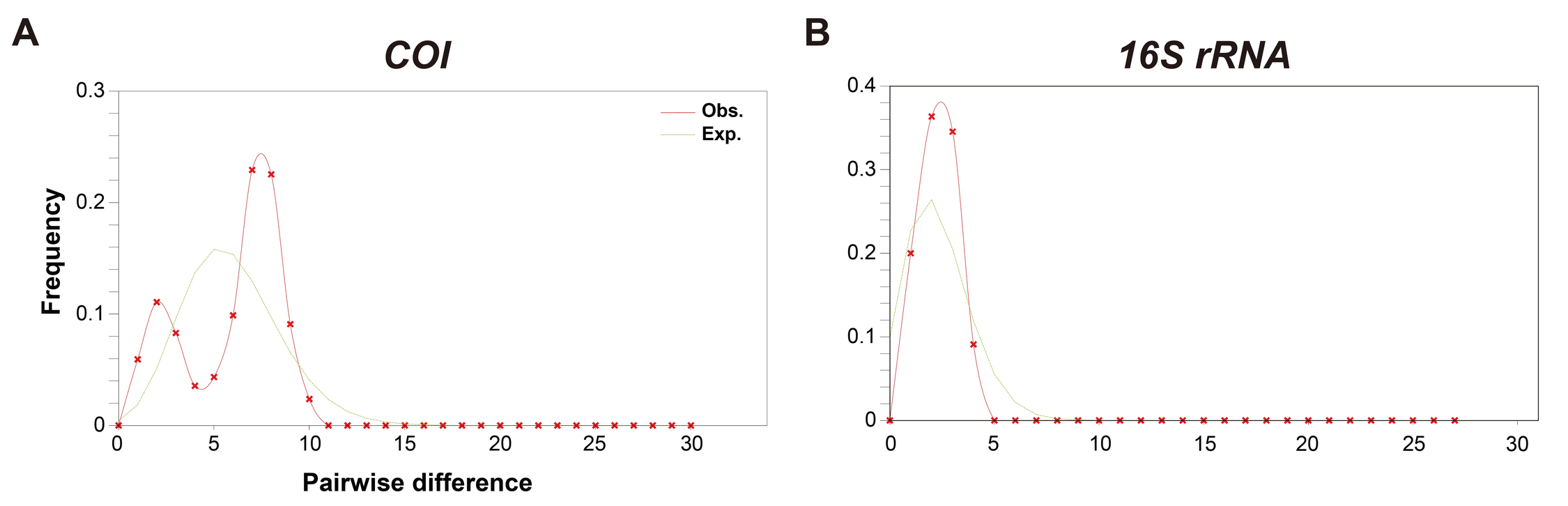

Supplement: S3 Fig — (TIF) [file pone.0288518.s003.tif]

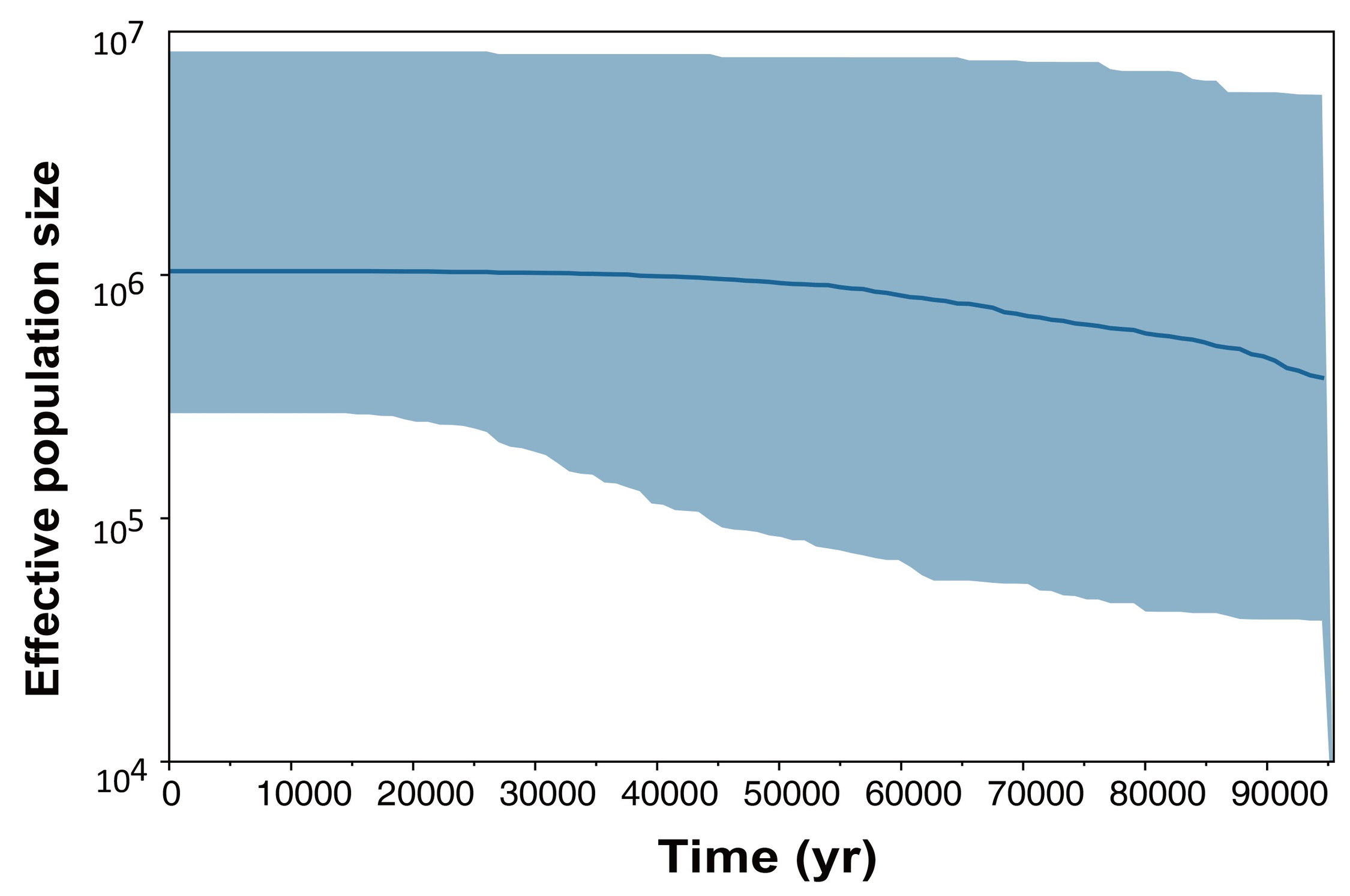

Supplement: S4 Fig — (TIF) [file pone.0288518.s004.tif]

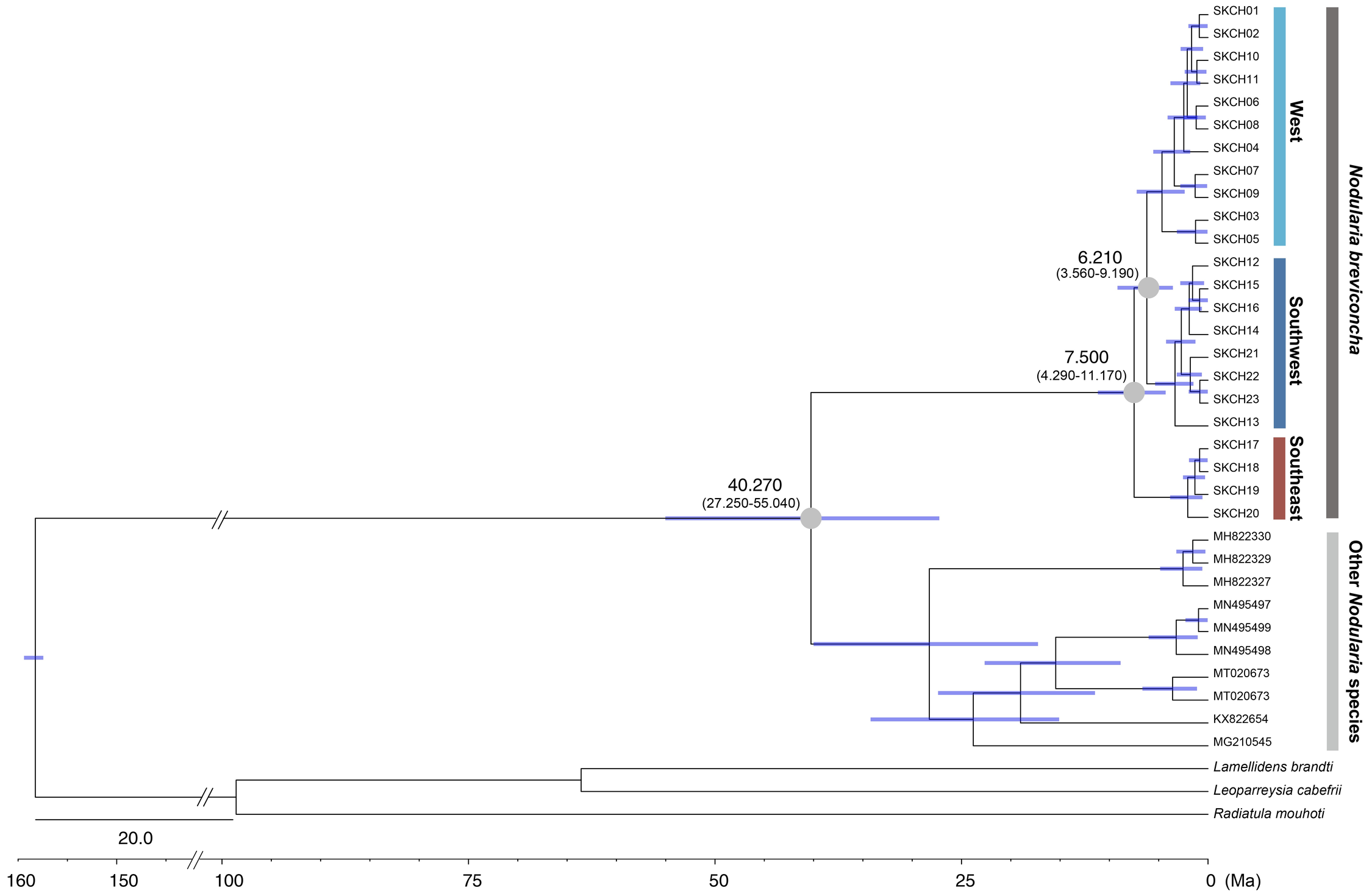

Supplement: S5 Fig — (TIF) [file pone.0288518.s005.tif]
