# Supplementary material for: The population genetic structure and phylogeographic dispersal of Nodularia breviconcha in the Korean Peninsula based on COI and 16S rRNA genes
Source: PLoS One. 2023 Jul 12;18(7):e0288518. doi: 10.1371/journal.pone.0288518 (PMC10337957; doi:10.1371/journal.pone.0288518)
Supplement: S1 Table — (DOCX) [file pone.0288518.s006.docx]

**S1 Table.** **List of collection sites and the number of *Nodularia breviconcha* individuals in each of the seven rivers.**

| **River** | **Abbreviation** | **Collection sites** | **N** | **References** |
| --- | --- | --- | --- | --- |
| Bukhan River | BH | Seo-myeon, Chuncheon-si, Gangwon, South Korea | 8 | Choi et al. (2020) |
|  |  | Naechon-myeon, Hongcheon-gun, Gangwon, South Korea | 21 | Choi et al. (2020) |
| Namhan River | NH | Gapcheon-myeon, Hoengseong-gun, Gangwon, South Korea | 20 | Choi et al. (2020) |
|  |  | Jucheon-myeon, Yeongwol-gun, Gangwon, South Korea | 23 | Choi et al. (2020) |
|  |  |  | 1 | This study |
| Geum River | GM | Geumsan-gun, Chungnam, South Korea | 1 | Choi et al. (2020) |
| Nakdong River | ND | Jiphyeon-myeon, Jinju-si, Gyeongnam, South Korea | 6 | This study |
|  |  | Dosan-myeon, Andong-si, Gyeongsbuk, South Korea | 21 | This study |
| Seomjin River | SJ | Sunchang-gun, Jeonbuk, South Korea | 1 | Choi et al. (2020) |
|  |  | Imsil-gun, Jeonbuk, South Korea | 1 | Choi et al. (2020) |
| Yeongsan River | YS | Geumjeong-myeon, Yeongam-gun, Jeonnam, South Korea | 5 | Choi et al. (2020) |
|  |  |  | 14 | This study |
| Tamjin River | TJ | Busan-myeon, Jangheung-gun, Jeonnam | 10 | This study |
|  |  |  | 3 | Choi et al. (2020) |
| Total | | | 135 |  |
