# Supplementary material for: The population genetic structure and phylogeographic dispersal of Nodularia breviconcha in the Korean Peninsula based on COI and 16S rRNA genes
Source: PLoS One. 2023 Jul 12;18(7):e0288518. doi: 10.1371/journal.pone.0288518 (PMC10337957; doi:10.1371/journal.pone.0288518)
Supplement: S2 Table — (DOCX) [file pone.0288518.s007.docx]

**S2 Table. Summary of sequence information of respective COI gene haplotypes from the *N. breviconcha* species in the present analyses.**

| **No.** | **Species** | **Country** | **Location** | **Accession No.** | **Haplotype** | **Reference** |
| --- | --- | --- | --- | --- | --- | --- |
| 01 | *Nodularia breviconcha* | South Korea | Bukhan River  Namhan River | MN495522 | SKCH01 | Choi et al. 2020 |
| 02 | *Nodularia breviconcha* | South Korea | Bukhan River  Namhan River | MN495523 | SKCH02 | Choi et al. 2020 |
| 03 | *Nodularia breviconcha* | South Korea | Bukhan River | MN495524 | SKCH03 | Choi et al. 2020 |
| 04 | *Nodularia breviconcha* | South Korea | Bukhan River | MN495531 | SKCH04 | Choi et al. 2020 |
| 05 | *Nodularia breviconcha* | South Korea | Bukhan River Namhan River | MN495525 | SKCH05 | Choi et al. 2020 |
| 06 | *Nodularia breviconcha* | South Korea | Bukhan River | MN495526 | SKCH06 | Choi et al. 2020 |
| 07 | *Nodularia breviconcha* | South Korea | Geum River | MN495532 | SKCH07 | Choi et al. 2020 |
| 08 | *Nodularia breviconcha* | South Korea | Namhan River | MN495527 | SKCH08 | Choi et al. 2020 |
| 09 | *Nodularia breviconcha* | South Korea | Namhan River | MN495528 | SKCH09 | Choi et al. 2020 |
| 10 | *Nodularia breviconcha* | South Korea | Namhan River | MN495529 | SKCH10 | Choi et al. 2020 |
| 11 | *Nodularia breviconcha* | South Korea | Namhan River | MN495530 | SKCH11 | Choi et al. 2020 |
| 12 | *Nodularia breviconcha* | South Korea | Seomjin River Tamjin River | MN495533 | SKCH12 | Choi et al. 2020 |
| 13 | *Nodularia breviconcha* | South Korea | Nakdong River  Seomjin River | MN495534 | SKCH13 | Choi et al. 2020 |
| 14 | *Nodularia breviconcha* | South Korea | Tamjin River | MN495535 | SKCH14 | Choi et al. 2020 |
| 15 | *Nodularia breviconcha* | South Korea | Yeongsan River | MN495536 | SKCH15 | Choi et al. 2020 |
| 16 | *Nodularia breviconcha* | South Korea | Yeongsan River | MN495537 | SKCH16 | Choi et al. 2020 |
| 17 | *Nodularia breviconcha* | South Korea | Nakdong River | OM283257 | SKCH17 | Present study |
| 18 | *Nodularia breviconcha* | South Korea | Nakdong River | OM283258 | SKCH18 | Present study |
| 19 | *Nodularia breviconcha* | South Korea | Nakdong River | OM283259 | SKCH19 | Present study |
| 20 | *Nodularia breviconcha* | South Korea | Nakdong River | OM283260 | SKCH20 | Present study |
| 21 | *Nodularia breviconcha* | South Korea | Tamjin River | OM283261 | SKCH21 | Present study |
| 22 | *Nodularia breviconcha* | South Korea | Yeongsan River | OM283262 | SKCH22 | Present study |
| 23 | *Nodularia breviconcha* | South Korea | Yeongsan River | OM283263 | SKCH23 | Present study |
| 24 | *Nodularia breviconcha* | South Korea | Seomjin River | MT020662 | SKCH12 | Lopes-Lima et al. 2020 |
| 25 | *Nodularia breviconcha* | South Korea | Han River | MT020663 | SKCH01 | Lopes-Lima et al. 2020 |
| 26 | *Nodularia breviconcha* | South Korea | Tamjin River | MT020666 | SKCH12 | Lopes-Lima et al. 2020 |
| 27 | *Nodularia breviconcha* | South Korea | Hyeonsan Stream | MT020667 | SKCH15 | Lopes-Lima et al. 2020 |
| 28 | *Nodularia breviconcha* | South Korea | Ungcheon Stream | MT020670 | SKCH07 | Lopes-Lima et al. 2020 |
| 29 | *Nodularia breviconcha* | South Korea | Namhan River | MT955592 | SKCH01 | Kim et al. 2020 |
