# Supplementary material for: The population genetic structure and phylogeographic dispersal of Nodularia breviconcha in the Korean Peninsula based on COI and 16S rRNA genes
Source: PLoS One. 2023 Jul 12;18(7):e0288518. doi: 10.1371/journal.pone.0288518 (PMC10337957; doi:10.1371/journal.pone.0288518)
Supplement: S3 Table — (DOCX) [file pone.0288518.s008.docx]

**S3 Table.** **Distribution of 23 COI gene haplotypes found in 135 individuals of *N. breviconcha* collected from the seven freshwater systems in the Korean Peninsula.**

| **Haplotype** | **Accession**  **No.** |  | **Freshwater systems** | | | | | | | **Total** |
| --- | --- | --- | --- | --- | --- | --- | --- | --- | --- | --- |
|  |  | **BH** | | **NH** | **GM** | **ND** | **SJ** | **YS** | **TJ** |  |
| SKCH01 | MN495522 | 12 | | 29 |  |  |  |  |  | 41 |
| SKCH02 | MN495523 | 1 | | 5 |  |  |  |  |  | 6 |
| SKCH03 | MN495524 | 1 | |  |  |  |  |  |  | 1 |
| SKCH04 | MN495531 | 1 | |  |  |  |  |  |  | 1 |
| SKCH05 | MN495525 | 11 | | 1 |  |  |  |  |  | 12 |
| SKCH06 | MN495526 | 3 | |  |  |  |  |  |  | 3 |
| SKCH07 | MN495532 |  | |  | 1 |  |  |  |  | 1 |
| SKCH08 | MN495527 |  | | 2 |  |  |  |  |  | 2 |
| SKCH09 | MN495528 |  | | 3 |  |  |  |  |  | 3 |
| SKCH10 | MN495529 |  | | 3 |  |  |  |  |  | 3 |
| SKCH11 | MN495530 |  | | 1 |  |  |  |  |  | 1 |
| SKCH12 | MN495533 |  | |  |  |  | 1 |  | 8 | 9 |
| SKCH13 | MN495534 |  | |  |  | 3 | 1 |  |  | 4 |
| SKCH14 | MN495535 |  | |  |  |  |  |  | 4 | 4 |
| SKCH15 | MN495536 |  | |  |  |  |  | 4 |  | 4 |
| SKCH16 | MN495537 |  | |  |  |  |  | 1 |  | 1 |
| SKCH17 | OM283257 |  | |  |  | 1 |  |  |  | 1 |
| SKCH18 | OM283258 |  | |  |  | 19 |  |  |  | 19 |
| SKCH19 | OM283259 |  | |  |  | 3 |  |  |  | 3 |
| SKCH20 | OM283260 |  | |  |  | 1 |  |  |  | 1 |
| SKCH21 | OM283261 |  | |  |  |  |  |  | 1 | 1 |
| SKCH22 | OM283262 |  | |  |  |  |  | 13 |  | 13 |
| SKCH23 | OM283263 |  | |  |  |  |  | 1 |  | 1 |
| **Total** | | **29** | | **44** | **1** | **27** | **2** | **19** | **13** | **135** |

BH, Bukhan River; GM, Geum River; NH, Namhan River; ND, Nakdong River; SJ, Seomjin River; YS, Yeongsan River; TJ, Tamjin River
