# Supplementary material for: The population genetic structure and phylogeographic dispersal of Nodularia breviconcha in the Korean Peninsula based on COI and 16S rRNA genes
Source: PLoS One. 2023 Jul 12;18(7):e0288518. doi: 10.1371/journal.pone.0288518 (PMC10337957; doi:10.1371/journal.pone.0288518)
Supplement: S4 Table — (DOCX) [file pone.0288518.s009.docx]

**S4 Table.** **Polymorphic sites in sequences of the 23 COI gene haplotypes of *N. breviconcha.***

| Haplotype | 0 | 0 | 0 | 0 | 1 | 1 | 2 | 2 | 2 | 2 | 2 | 2 | 2 | 3 | 3 | 3 | 3 | 3 | 3 | 3 | 4 | 4 | 4 | 4 | 4 | 4 | 4 | 4 | 5 |
| --- | --- | --- | --- | --- | --- | --- | --- | --- | --- | --- | --- | --- | --- | --- | --- | --- | --- | --- | --- | --- | --- | --- | --- | --- | --- | --- | --- | --- | --- |
|  | 0 | 3 | 5 | 8 | 3 | 6 | 2 | 2 | 3 | 4 | 5 | 8 | 8 | 2 | 4 | 6 | 7 | 8 | 9 | 9 | 5 | 6 | 6 | 7 | 7 | 8 | 8 | 9 | 1 |
|  | 3 | 9 | 4 | 8 | 2 | 2 | 2 | 5 | 1 | 6 | 8 | 2 | 8 | 7 | 5 | 9 | 9 | 4 | 0 | 3 | 3 | 2 | 4 | 4 | 9 | 0 | 3 | 5 | 3 |
| SKCH01 | A | C | A | G | G | C | T | T | C | A | A | T | C | A | C | G | C | C | T | C | C | T | G | A | A | T | T | G | C |
| SKCH02 | • | • | • | • | • | • | • | • | • | G | • | • | • | • | • | • | • | • | • | • | • | • | • | • | • | • | • | • | • |
| SKCH03 | • | • | T | • | • | • | • | • | • | • | • | • | • | • | T | • | • | • | • | • | • | • | • | • | • | • | • | • | • |
| SKCH04 | • | • | • | • | • | • | • | C | • | • | • | • | • | • | • | • | • | A | • | • | • | • | • | • | • | • | • | • | • |
| SKCH05 | • | • | T | • | • | • | • | • | • | • | • | • | • | • | • | • | • | • | • | • | • | • | • | • | • | • | • | • | • |
| SKCH06 | • | • | • | • | • | • | • | • | A | • | • | • | • | • | • | • | • | • | • | • | • | • | • | • | • | • | • | • | • |
| SKCH07 | • | • | • | • | A | • | C | • | • | • | • | • | • | • | • | A | • | • | C | • | • | • | • | G | • | • | C | • | • |
| SKCH08 | • | • | • | • | • | • | • | • | • | • | • | • | • | • | • | • | • | • | • | • | G | • | • | • | • | • | • | • | • |
| SKCH09 | • | • | • | • | A | • | C | • | • | • | • | • | • | • | • | • | • | • | • | • | • | • | • | G | • | • | C | • | • |
| SKCH10 | • | • | • | A | • | • | • | • | • | • | • | • | • | • | • | • | • | • | • | • | • | • | • | • | • | • | • | • | • |
| SKCH11 | • | • | • | • | • | • | • | • | • | • | • | • | • | • | • | • | A | • | • | • | • | • | • | • | • | • | • | • | • |
| SKCH12 | • | • | T | • | • | • | G | • | • | • | • | • | • | G | • | A | • | • | • | • | • | • | • | G | • | • | • | • | • |
| SKCH13 | • | • | T | • | • | • | G | • | • | • | • | C | • | G | • | A | • | • | • | • | • | • | • | G | • | • | • | A | • |
| SKCH14 | • | • | T | • | • | • | G | • | • | • | • | • | • | G | • | A | • | • | • | • | • | • | • | G | • | • | • | • | T |
| SKCH15 | • | T | T | • | • | • | G | • | • | • | • | • | • | G | • | A | • | • | • | • | • | • | • | G | • | • | • | • | • |
| SKCH16 | G | T | T | • | • | • | G | • | • | • | • | • | • | G | • | A | • | • | • | • | • | • | • | G | • | • | • | • | • |
| SKCH17 | • | • | • | • | • | • | • | • | • | • | • | • | T | • | • | A | • | • | • | T | • | • | A | G | G | • | • | • | • |
| SKCH18 | • | • | T | • | • | • | • | • | • | • | • | • | T | • | • | A | • | • | • | T | • | • | A | G | G | • | • | • | • |
| SKCH19 | • | • | T | • | • | • | • | • | • | • | • | • | T | • | • | A | • | • | • | T | • | • | A | G | G | C | • | • | • |
| SKCH20 | • | • | T | • | • | • | • | • | • | • | G | • | T | • | • | A | • | • | • | T | • | • | A | G | G | • | • | • | • |
| SKCH21 | • | • | T | • | • | A | G | • | • | • | • | • | T | G | • | A | • | • | • | • | • | • | • | G | • | • | • | • | • |
| SKCH22 | • | • | T | • | • | • | G | • | • | • | • | • | • | G | • | A | • | • | • | A | • | • | • | G | • | • | • | • | • |
| SKCH23 | • | • | T | • | • | • | G | • | • | • | • | • | • | G | • | A | • | • | • | A | • | C | • | G | • | • | • | • | • |

Digits at the top of the figure indicate nucleotide positions. Dots (․) represent the same bases with those of the first-line haplotype SKCH01.
