# Supplementary material for: The population genetic structure and phylogeographic dispersal of Nodularia breviconcha in the Korean Peninsula based on COI and 16S rRNA genes
Source: PLoS One. 2023 Jul 12;18(7):e0288518. doi: 10.1371/journal.pone.0288518 (PMC10337957; doi:10.1371/journal.pone.0288518)
Supplement: S5 Table — (DOCX) [file pone.0288518.s010.docx]

**S5 Table.** **Summary of sequence information of respective 16S rRNA gene haplotypes from the *N. breviconcha* species used in the present analyses.**

| **No.** | **Species** | **Country** | **Location** | **Accession No.** | **Haplotype** | **Reference** |
| --- | --- | --- | --- | --- | --- | --- |
| 01 | *Nodularia breviconcha* | South Korea | Bukhan River  Namhan River | MN495488 | SKSH01 | Choi et al. 2020 |
| 02 | *Nodularia breviconcha* | South Korea | Bukhan River | MN495489 | SKSH02 | Choi et al. 2020 |
| 03 | *Nodularia breviconcha* | South Korea | Bukhan River  Nakdong River | MN495491 | SKSH03 | Choi et al. 2020 |
| 04 | *Nodularia breviconcha* | South Korea | Namhan River | MN495492 | SKSH04 | Choi et al. 2020 |
| 05 | *Nodularia breviconcha* | South Korea | Namhan River | MN495493 | SKSH05 | Choi et al. 2020 |
| 06 | *Nodularia breviconcha* | South Korea | Yeongsan River  Tamjin River | OM283265 | SKSH06 | Present study |
| 07 | *Nodularia breviconcha* | South Korea | Yeongsan River  Tamjin River | OM283266 | SKSH07 | Present study |
| 08 | *Nodularia breviconcha* | South Korea | Yeongsan River | OM283267 | SKSH08 | Present study |
| 09 | *Nodularia breviconcha* | South Korea | Yeongsan River | OM283268 | SKSH09 | Present study |
| 10 | *Nodularia breviconcha* | South Korea | Yeongsan River | OM283269 | SKSH10 | Present study |
| 11 | *Nodularia breviconcha* | South Korea | Seomjin River | OM283270 | SKSH11 | Present study |
| 12 | *Nodularia breviconcha* | South Korea | Namhan River | MF314443 | SKSH01 | Kim et al. 2020 |
