# Supplementary material for: The population genetic structure and phylogeographic dispersal of Nodularia breviconcha in the Korean Peninsula based on COI and 16S rRNA genes
Source: PLoS One. 2023 Jul 12;18(7):e0288518. doi: 10.1371/journal.pone.0288518 (PMC10337957; doi:10.1371/journal.pone.0288518)
Supplement: S6 Table — (DOCX) [file pone.0288518.s011.docx]

**S6 Table.** **Distribution of 11 16S rRNA gene haplotypes found in 131 individuals of *N. breviconcha* collected from the six freshwater systems in the Korean Peninsula.**

| **Haplotype** | **Accession**  **No.** |  | **Freshwater systems** | | | | | **Total** |
| --- | --- | --- | --- | --- | --- | --- | --- | --- |
|  |  | **BH** | **NH** | **ND** | **SJ** | **YS** | **TJ** |  |
| SKSH01 | MN495488 | 26 | 42 |  |  |  |  | 68 |
| SKSH02 | MN495489 | 2 |  |  |  |  |  | 2 |
| SKSH03 | MN495491 | 1 |  | 24 |  |  |  | 25 |
| SKSH04 | MN495492 |  | 1 |  |  |  |  | 1 |
| SKSH05 | MN495493 |  | 1 |  |  |  |  | 1 |
| SKSH06 | OM283265 |  |  | 3 |  |  |  | 3 |
| SKSH07 | OM283266 |  |  |  |  | 4 | 12 | 16 |
| SKSH08 | OM283267 |  |  |  |  | 4 |  | 4 |
| SKSH09 | OM283268 |  |  |  |  | 9 |  | 9 |
| SKSH10 | OM283269 |  |  |  |  | 1 |  | 1 |
| SKSH11 | OM283270 |  |  |  | 1 |  |  | 1 |
| **Total** | | **29** | **44** | **27** | **1** | **18** | **12** | **131** |

BH, Bukhan River; NH, Namhan River; ND, Nakdong River; SJ, Seomjin River; YS, Yeongsan River; TJ, Tamjin River
