# Supplementary material for: The population genetic structure and phylogeographic dispersal of Nodularia breviconcha in the Korean Peninsula based on COI and 16S rRNA genes
Source: PLoS One. 2023 Jul 12;18(7):e0288518. doi: 10.1371/journal.pone.0288518 (PMC10337957; doi:10.1371/journal.pone.0288518)
Supplement: S7 Table — (DOCX) [file pone.0288518.s012.docx]

**S7 Table.** **Polymorphic sites in sequences of the 11 16S rRNA gene haplotypes of *N. breviconcha.***

| Haplotype | | 0 | 1 | 1 | 1 | 2 | 2 | | 2 | 2 |
| --- | --- | --- | --- | --- | --- | --- | --- | --- | --- | --- |
|  |  | 7 | 1 | 7 | 9 | 2 | 4 | | 5 | 7 |
|  |  | 1 | 8 | 5 | 3 | 9 | 7 | | 7 | 5 |
| SKSH01 | | A | A | C | G | A | T | | A | G |
| SKSH02 | | • | • | • | • | • | A | | • | • |
| SKSH03 | | • | • | • | A | • | • | | • | • |
| SKSH04 | | • | • | • | • | G | • | | • | • |
| SKSH05 | | • | • | T | • | • | • | | C | • |
| SKSH06 | | G | • | • | • | • |  | | C | • |
| SKSH07 | | • | • | • | • | • |  | | C | • |
| SKSH08 | | • | • | • | • | • |  | | C | A |
| SKSH09 | | • | G | • | • | • |  | | C | A |
| SKSH10 | | • | • | • | • | G |  | | C | A |
| SKSH11 | | • | • | • | A | • | • | | C | • |
| Digits at the top of the figure indicate nucleotide positions. Dots (․) represent the same bases with those of the first-line haplotype SKSH01. | | | | | | | | | | |
|  | | | | | | |  |  |  |  |
