# Supplementary material for: The population genetic structure and phylogeographic dispersal of Nodularia breviconcha in the Korean Peninsula based on COI and 16S rRNA genes
Source: PLoS One. 2023 Jul 12;18(7):e0288518. doi: 10.1371/journal.pone.0288518 (PMC10337957; doi:10.1371/journal.pone.0288518)
Supplement: S8 Table — (DOCX) [file pone.0288518.s013.docx]

**S8 Table.** **Pairwise *F_ST_* values estimated with** **the five populations of *N. breviconcha* based on 16S rRNA gene sequences in the Korean Peninsula.**

| Pop | BH | NH | ND | YS | TJ |
| --- | --- | --- | --- | --- | --- |
| BH | 0 |  |  |  |  |
| NH | 0.01729 | 0 |  |  |  |
| ND | **0.65011^***^** | **0.71676^***^** | 0 |  |  |
| YS | 0.79445 | 0.82431 | 0.75823 | 0 |  |
| TJ | **0.87307^***^** | **0.89746^***^** | **0.78368^***^** | **0.57166^*^** | 0 |
