# Supplementary material for: The population genetic structure and phylogeographic dispersal of Nodularia breviconcha in the Korean Peninsula based on COI and 16S rRNA genes
Source: PLoS One. 2023 Jul 12;18(7):e0288518. doi: 10.1371/journal.pone.0288518 (PMC10337957; doi:10.1371/journal.pone.0288518)
Supplement: S9 Table — (DOCX) [file pone.0288518.s014.docx]

**S9 Table.** **Analysis of molecular variance (AMOVA) performed based on the COI gene sequences of 135 *N. breviconcha* individuals*.***

| **Grouping** | **Source of Variation** | **Degree of freedom** | **Sum of squares** | **Variance components** | **Percentage of variation** | ***p*** |
| --- | --- | --- | --- | --- | --- | --- |
| Three lineages  (West vs. Southwest vs. Southeast) | Among groups | 2 | 262.760 | 3.067 | 75.590 | <0.05 |
|  | Among populations within groups | 4 | 18.641 | 0.289 | 7.130 | <0.001 |
|  | Within populations | 128 | 89.743 | 0.701 | 17.280 | <0.001 |
