# Supplementary material for: The population genetic structure and phylogeographic dispersal of Nodularia breviconcha in the Korean Peninsula based on COI and 16S rRNA genes
Source: PLoS One. 2023 Jul 12;18(7):e0288518. doi: 10.1371/journal.pone.0288518 (PMC10337957; doi:10.1371/journal.pone.0288518)
Supplement: S10 Table — (DOCX) [file pone.0288518.s015.docx]

**S10 Table. Spatial analysis of molecular variance (SAMOVA) performed based on the 16S rRNA gene sequences of 131 individuals with the highest *F_CT_* (maximum variation between defined populations).**

| **K value** | ***F_CT_*** | ***F_SC_*** | ***F_ST_*** | **Grouping** |
| --- | --- | --- | --- | --- |
| 2 | 0.66287 | **0.60636^***^** | **0.66287^***^** | (BH-NH-ND-SJ-TJ)(YS) |
| 3 | 0.66218 | **0.59866^***^** | **0.86442^***^** | (BH-NH-ND-SJ)(TJ)(YS) |
| 4 | **0.78068^*^** | 0.05519 | **0.79279^***^** | (BH-NH)(ND-SJ)(TJ)(YS) |
| 5 | 0.79429 | -0.00797 | **0.79265^***^** | (BH-NH)(ND)(SJ)(TJ)(YS) |

Statistically significant values are written in bold: *P < 0.05; **P<0.01; ***P<0.001.
