# Supplementary material for: The population genetic structure and phylogeographic dispersal of Nodularia breviconcha in the Korean Peninsula based on COI and 16S rRNA genes
Source: PLoS One. 2023 Jul 12;18(7):e0288518. doi: 10.1371/journal.pone.0288518 (PMC10337957; doi:10.1371/journal.pone.0288518)
Supplement: S11 Table — (DOCX) [file pone.0288518.s016.docx]

| Gene | Detailed group | r | SSD |
| --- | --- | --- | --- |
| COI | West | **0.117^***^** | **0.014^***^** |
|  | Southeast | 0.184 | 0.004 |
|  | Southwest | 0.004 | 0.050 |
|  | Total | 0.051 | **0.044^*^** |
| 16S rRNA | Total | 0.060 | 0.005 |

**S11 Table.** **Raggedness index (r) and sum of squares deviation (SSD) estimated from the mismatch distribution analysis based on COI and 16S rRNA genes for *N. breviconcha* on the Korean Peninsula.**

Statistically significant values are indicated in bold: *P < 0.05, **P<0.01, ***P<0.001.
