# Supplementary material for: The population genetic structure and phylogeographic dispersal of Nodularia breviconcha in the Korean Peninsula based on COI and 16S rRNA genes
Source: PLoS One. 2023 Jul 12;18(7):e0288518. doi: 10.1371/journal.pone.0288518 (PMC10337957; doi:10.1371/journal.pone.0288518)
Supplement: S12 Table — (DOCX) [file pone.0288518.s017.docx]

**S12 Table.** **The information of primers used for PCR amplification of CO1 and 16S rRNA genes*.***

| **Gene** | **Primer** | **Sequence (5´→3´)** |
| --- | --- | --- |
| **CO1** | LCO22me2^1)^ | GGT CAA CAA AYC ATA ARG ATA TTG G |
|  | HCO700dy2^1)^ | TCA GGG TGA CCA AAA AAY CA |
| **16S rRNA** | 16Sar-L-myt^2)^ | CGA CTG TTT AAC AAA AAC AT |
|  | 16Sbr-H-myt^2)^ | CCG TTC TGA ACT CAG CTC ATG T |
| ^1)^Walker *et al*. (2006), ^2)^Lydeard *et al*. (1996). | | |
