# Supplementary material for: The population genetic structure and phylogeographic dispersal of Nodularia breviconcha in the Korean Peninsula based on COI and 16S rRNA genes
Source: PLoS One. 2023 Jul 12;18(7):e0288518. doi: 10.1371/journal.pone.0288518 (PMC10337957; doi:10.1371/journal.pone.0288518)
Supplement: S13 Table — (DOCX) [file pone.0288518.s018.docx]

**S13 Table. Summary of sequence information of outgroups used in the time-divergence tree using the BEAST 2.6.0.**

| **No.** | **Species** | **Country** | **Accession No.** | **Reference** |
| --- | --- | --- | --- | --- |
| 01 | *Nodularia douglasiae* | South Korea | MN495497 | Choi et al. 2020 |
| 02 | *Nodularia douglasiae* | South Korea | MN495489 | Choi et al. 2020 |
| 03 | *Nodularia douglasiae* | South Korea | MN495491 | Choi et al. 2020 |
| 04 | *Nodularia nipponensis* | Japan | MT020673 | Lopes-lima et al. 2020 |
| 05 | *Nodularia nipponensis* | Japan | MT020674 | Lopes-lima et al. 2020 |
| 06 | *Nodularia nuxpersicae* | Vietnam | KX822654 | Lopes-lima et al. 2020 |
| 07 | *Nodularia* sp*.*1 | China | MG210545 | Liu et al. 2017 |
| 08 | *Nodularia* sp*.*2 | China | MH822327 | Liu et al. 2019 |
| 09 | *Nodularia* sp*.*2 | China | MH822329 | Liu et al. 2019 |
| 10 | *Nodularia* sp*.*2 | China | MH822330 | Liu et al. 2019 |
| 11 | *Lamellidens brandti* | Myanmar | MF352224 | Bolotov et al. 2017 |
| 12 | *Leoparreysia cabefrii* | Myanmar | MF352251 | Bolotov et al. 2017 |
| 13 | *Radiatula mouhoti* | Myanmar | MF352257 | Bolotov et al. 2017 |
